# Oropouche virus efficiently replicates and is immunostimulatory in vivo in nonhuman primate species

**DOI:** 10.1126/sciadv.adx9405

**Published:** 2025-09-17

**Authors:** Debra S. Yee, Jonathan P. Davies, Laura A. VanBlargan, Andrew R. Rahmberg, Katherine E. Burgomaster, Kelsie Brooks, Jacob K. Flynn, Byron Shue, Alexandra M. Ortiz, Paul Schaughency, Sonja M. Best, Theodore C. Pierson, Jason M. Brenchley

**Affiliations:** ^1^ Barrier Immunity Section, Laboratory of Viral Diseases, National Institute of Allergy and Infectious Diseases, NIH, Bethesda, MD, USA.; ^2^ Arbovirus Immunity Section, Vaccine Research Center, NIAID, NIH, Bethesda, MD, USA.; ^3^ Innate Immunity and Pathogenesis Section, Laboratory of Neurological Infections and Immunity, NIAID, NIH, Hamilton, MT, USA.; ^4^ Integrated Data Sciences Section, Research Technologies Branch, NIAID, NIH, Bethesda, MD, USA.

## Abstract

An Oropouche virus (OROV) outbreak occurred in South America in 2024. The pathogenic potential and host immunological response of this emerging virus are largely unknown, as animal models have been poorly explored. We infected nonhuman primate (NHP) species with OROV and followed viral replication dynamics and subsequent innate and adaptive immunological responses. OROV efficiently replicated in pigtail macaques, rhesus macaques, and vervet African green monkeys. OROV also replicated in sabeus African green monkey, albeit at lower levels than other hosts. OROV RNA was detected in plasma and nasal swabs and infection-induced high levels of innate inflammation, type I interferon gene signatures, immunoglobulin M–positive B cell expansion, high titers of neutralizing antibodies, and detectable frequencies of OROV-specific T cells. Prior infection was protective against reinfection up to 524 days post–initial infection, demonstrating possible protective immunity induction against OROV infection. These data suggest that multiple NHP species are appropriate models for OROV infection and the development of therapeutics and vaccinations.

## Teaser

OROV replicates in experimental NHP species.

## INTRODUCTION

Over the course of the past decade, there have been several viral outbreaks that have had a substantial impact on human health. Emerging arboviruses are poised for rapid geographical expansion, as witnessed by the Zika virus (ZIKV) in the Americas, Japanese encephalitis virus in Australia, and, recently, Oropouche virus (OROV) in Latin America (*1*–*4*). OROV is a member of the Peribunyaviridae family, a group of segmented, negative sense, single-stranded RNA arboviruses that includes viruses with considerable pandemic potential (*5*). Of the more than 300 viruses within the Peribunyaviridae family, more than 50 are known to cause human disease (*6*–*8*). OROV is not only predominantly spread by midge bites but has also been found in several mosquito species and has a wide range of vertebrate hosts (*7*, *9*). In humans, OROV can cause acute febrile disease with rare neurological and encephalitis symptomologies (*7*, *10*, *11*) and may cause stillbirths and neurological defects in babies infected in utero (*11*, *12*). Genetic reassortment of OROV has also been observed (*13*). Deforestation is suspected to contribute to the spread of OROV and other arboviruses (*14*); however, OROV is a poorly studied arbovirus (*11*).

Animal models of viral infections are critically important for understanding cells targeted by the virus in vivo, mechanisms of disease pathogenesis, and the development of efficacious vaccines and antivirals (*15*). A common animal model of viral infections involves genetically modifying mice to remove signaling through type I interferons (IFNs) (*16*). Virally encoded mechanisms of IFN antagonism that are important for establishing infection in humans can be host species restricted and do not counteract murine antiviral responses, and, as a result, wild-type mice are often resistant to infections (*16*). Thus, mice genetically engineered to be deficient for IFN signaling pathways have been used as small animal models of OROV pathogenesis (*17*, *18*). However, the loss of critical innate immune responses precludes a complete understanding of antiviral innate and adaptive immunity. Other critically important animal models of human disease include Asian and African nonhuman primate (NHP) species. Given the genetic similarity to humans, differences in IFN-mediated resistances to viral infection (as observed in mice) have not been reported (*15*). The most common NHP species used is *Macaca mulatta* of Indian origin [rhesus macaque (RM)], with less commonly used species within the *Macaca* genus, including *Macaca nemestrina* [pigtail macaque (PTM)] and *Macaca fascicularis* (cynomolgus macaque) (*15*). Of African NHP species, the most commonly studied is the sabeus African green monkey (AGMsab), *Chlorocebus sabeus*, which appears to be more permissive to infection with respiratory viruses than Asian NHP species (*15*).

Here, we explore in vivo OROV replication, clinical symptomologies, and immune responses (innate and adaptive) in two species of Asian macaques (RM and PTM) and two species of AGMs [AGMsab and vervet (AGMver)]. We find that OROV efficiently replicates and induces innate and adaptive immunity in PTM, RM, AGMsab, and AGMver without obvious clinical manifestations of infection. These data demonstrate that NHP species are appropriate for the development and assessment of antiviral countermeasures aimed at reducing OROV replication in vivo.

## RESULTS

### OROV replication in NHP species in vivo

**Fig. 1.**
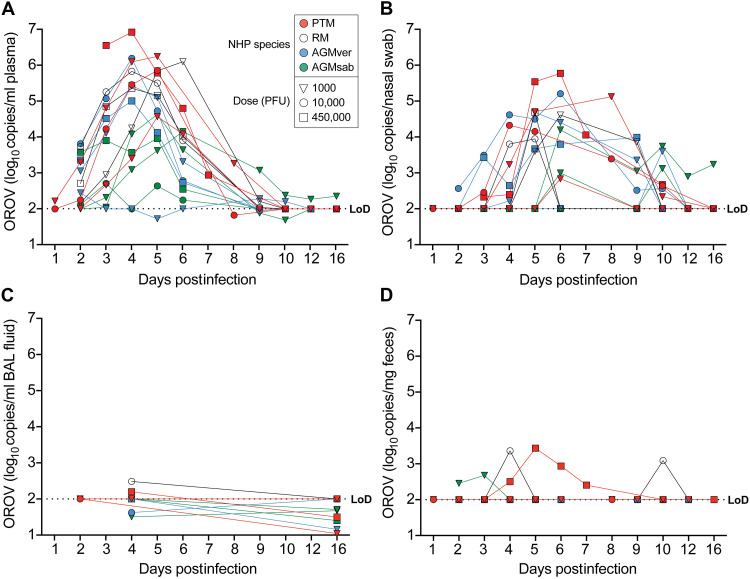
OROV RNA levels in vivo. Longitudinal levels of OROV RNA in plasma (**A**), nasal swabs (**B**), BAL (**C**), and fecal samples (**D**) in PTMs (red), RMs (open), AGMvers (blue), and AGMsabs (green). Shapes indicate inoculum dose. Horizontal lines represent limit of detection (LoD).

To determine whether different NHP species were susceptible to OROV infection and to understand the kinetics of viral replication, we examined OROV replication in four PTMs, four AGMvers, four AGMsabs, and six RM (Table 1). We initially injected 450,000 plaque-forming units (PFU) subcutaneously into one male PTM. We then measured OROV RNA in plasma, bronchoalveolar lavage (BAL) fluid, nasal swabs, and fecal samples between days 3 and 16 postinfection (Fig. 1, A to D, red lines). OROV RNA was quantified relative to both a plasmid containing the amplicon (*19*) and serial dilutions of a titered viral stock grown in vitro. We found high levels (3 million copies/ml) of OROV RNA (7000 PFU equivalents) at day 3 postinfection that increased at day 4 (8 million copies) and then consistently decreased daily. Since it was clear that OROV replicated in this animal, we next de-escalated the amount of virus in the inoculum in three additional PTMs. One animal received 10,000 PFU, and two animals received only 1000 PFU; we sampled animals days 1 to 5 postinfection. Viral RNA was detectable in plasma as early as 2 days postinfection, maximized at days 3 to 4 postinfection, and became undetectable around 9 days postinfection. Animals had similar magnitude and duration of viremia irrespective of the inoculum dose (Fig. 1A).

**Table 1.** Study animals. Demographic data and OROV inoculum dosages used for in vivo studies. Dashes indicate ages unknown. M, male; F, female.

Animal ID	Age (years)	Sex	Dose (PFU)
PTM1	18.8	M	1000
PTM2	18.1	M	1000
PTM3	17.8	M	10,000
PTM4	16.7	M	450,000
RM1	9.8	M	1000
RM2	12.4	F	1000
RM3	15.6	F	1000
RM4	10.8	M	1000
RM5	9.6	M	10,000
RM6	18.8	M	450,000
AGMver1	13.4	M	1000
AGMver2	9.2	M	1000
AGMver3	5.8	M	10,000
AGMver4	5.6	M	450,000
AGMsab1	–	M	1000
AGMsab2	–	M	1000
AGMsab3	–	F	10,000
AGMsab4	–	F	450,000

We next explored how promiscuous OROV replication might be across multiple NHP species. We infected six RMs, four AGMsabs, and four AGMvers with the same three doses used in studies with PTMs (Fig. 1A). As with PTM, OROV viral RNA was detected as early as day 2 postinfection, peaked at days 3 to 5 postinfection, and became undetectable by 9 days. Moreover, as with the PTMs, the viral titer used for inoculation did not markedly change the kinetics or the magnitude of viremia. While peak levels of OROV RNA in RM and AGMver were similar to those seen in PTM (>10^6^), OROV RNA levels in AGMsab tended to be lower, peaking almost two orders of magnitude lower (~10^4^). Of the six RM we infected, four RM were infected with the lowest dose of 1000-PFU OROV, although no detectable viremia occurred in three RMs infected with 1000 PFU and no evidence of immunological responses. Low levels of OROV RNA were observed in an AGM that was infected with 1000 PFU (Fig. 1A). Thus, 10,000 PFU was the lowest inoculum we found could reliably lead to productive infection of NHPs. Together, it is clear that OROV has a broad range of primate hosts capable of supporting acute systemic viremia dissemination.

We next explored the dissemination of OROV RNA to other anatomic sites, including the upper air ways (nasal swabs; Fig. 1B), lower air ways (BAL fluid; Fig. 1C), and the gastrointestinal tract (fecal samples; Fig. 1D). Detectable levels of viral RNA were commonly found in the upper airways with kinetics that roughly mirrored those observed in the plasma (Fig. 1B). Levels of viral RNA were lower and mostly undetected in BAL samples (Fig. 1C) or feces (Fig. 1D). Neurological symptomologies can be associated with more severe OROV infection (*20*). Therefore, we sampled cerebrospinal fluid (CSF) of OROV-infected animals twice during the time frame when OROV was detectable in plasma (Table 2). Obtaining CSF from NHPs is often difficult and contamination with peripheral blood is common and may contribute to positive sampling of OROV RNA within CSF. Cytology was performed to estimate the degree to which peripheral blood was contaminating the CSF. We also determined the level of plasma contamination that would need to be present to account for the OROV RNA within the CSF (based on plasma OROV RNA concentrations). Low levels of OROV RNA were detected, and there were occasional signs of lymphocytic infiltration not consistent with peripheral blood contamination. There were only rare instances where plasma contamination was highly unlikely to account for all the OROV RNA detected (Table 2). Thus, neuroinvasion by OROV was possible in at least three of the NHPs we studied. This was more common in the AGMsab than other NHP species.

**Table 2.** OROV RNA copies in CSF. Viral loads from CSF and plasma with corresponding red blood cell (RBC) counts. Dashes indicate data not applicable or not available. ND, not detected.

			Viral load (copies/ml)		
Animal ID	Days postinfection	RBC (per μl of CSF)	CSF	Plasma	% Plasma contamination to account for all CSF OROV RNA
PTM1	−11	–	ND	ND	–
	−4	–	ND	ND	–
	2	–	ND	ND	–
	6	–	ND	ND	–
	12	–	ND	ND	–
PTM2	−5	19,980	ND	ND	–
	5	35	312	1,700,000	0.018
PTM3	−5	–	ND	ND	–
	1	3200	ND	ND	–
	5	225	197	700,000	0.028
PTM4	−3	0	ND	ND	–
	3	3800	1493	3,500,000	0.043
	7	–	159	870	18
	12	7500	ND	ND	–
RM4	−11	284	ND	ND	–
	−4	39	ND	ND	–
	2	1977	ND	ND	–
	12	0	ND	ND	–
RM5	−11	2	ND	ND	–
	2	95,400	ND	ND	–
	12	–	ND	ND	–
RM6	−11	–	ND	ND	–
	12	1336	ND	ND	–
AGMver1	−8	–	ND	ND	–
	2	24	ND	ND	–
	6	4	ND	ND	–
	12	594	ND	ND	–
AGMver2	−11	103,950	ND	ND	–
	−4	5816	ND	ND	–
	2	445	ND	ND	–
	6	5370	46	2000	2.2
	12	–	ND	ND	–
AGMver3	−11	23	ND	ND	–
	3	–	56,392	120,000	48
	12	–	ND	ND	–
AGMver4	−11	–	ND	ND	–
	−4	34,897	ND	ND	–
	2	106,920	77	2300	3.4
	6	1108	188	540	35
	12	–	ND	ND	–
AGMsab1	−11	10,530	ND	ND	–
	−4	6025	ND	ND	–
	2	831	ND	ND	–
	6	6534	ND	ND	–
	12	–	341	ND	–
AGMsab2	−8	–	ND	ND	–
	2	990	ND	ND	–
	6	27	ND	ND	–
	12	–	1340	49	2700
AGMsab3	−8	–	ND	ND	–
	2	3564	ND	ND	–
	6	–	121	180	69
	12	1	47	ND	–
AGMsab4	−8	–	ND	ND	–
	2	–	ND	ND	–
	6	71,280	268	360	75

To determine whether the viral RNA we measured corresponded to replication-competent virus, we attempted virus recovery from plasma samples by coculturing with Vero cells. Replication competent virus was recovered from plasma of PTM sampled at day 4 postinfection.

### Symptomology

The clinical manifestations following OROV infection can range from asymptomatic to acute fever, headache, myalgia, arthralgia, anorexia, dizziness, chills, nausea, vomiting, diarrhea, epigastric pain, photophobia, or retro-orbital pain (*21*). To determine whether OROV infection was associated with any clinical manifestations in NHPs, we monitored animals for quality of stool, appetite, behavior, respiratory symptoms, skin appearance, nasal discharge, ocular signs, hydration, blood oxygen saturation, respiratory rates, pulse, weight, and rectal temperature. Observations were made daily for 2 days before infection and then daily for 3 weeks postinfection. We also monitored blood chemistries (blood glucose, liver enzymes, kidney function, proteins, and phosphorus) and complete blood counts 2 days before infection and then every day that blood was obtained to measure OROV viral RNA. No adverse events were observed for clinical symptomologies, blood chemistries, or complete blood counts relative to uninfected animals that are anesthetized daily for sampling (fig. S1). While none of the animals (*n* = 15) with detectable plasma OROV RNA had any observable adverse events, it is unclear how many asymptomatic cases of OROV infection occur in humans (*20*). Therefore, it is also unclear whether OROV infection of NHPs recapitulates the incidence of symptomologies in humans.

### Immune responses

**Fig. 2.**
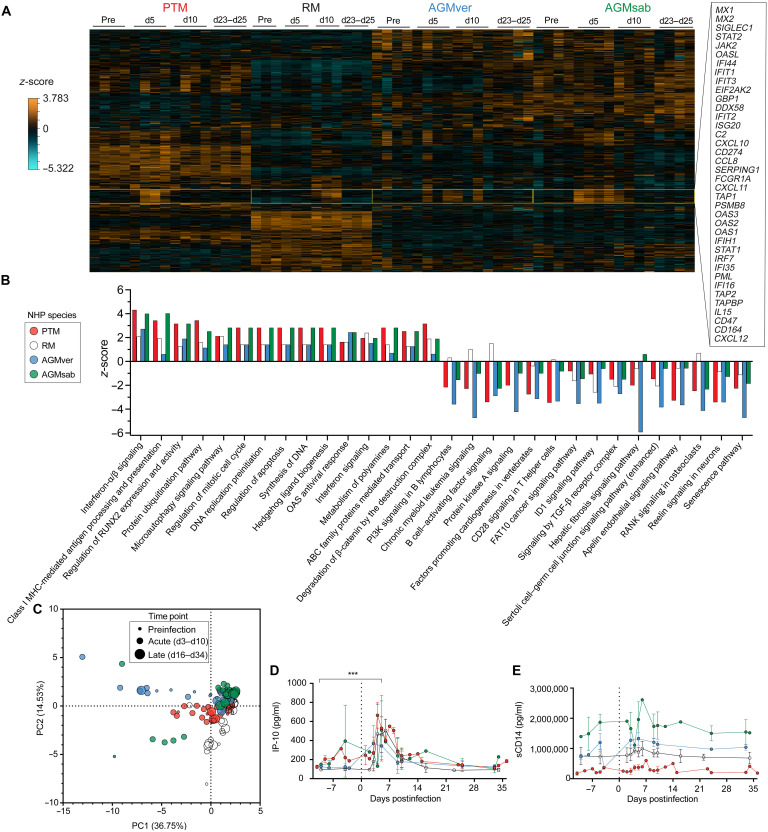
Innate immune activation after OROV infection in vivo. Longitudinal expression levels of approximately 800 genes of immunological interest by NanoString analysis in PBMCs in PTM, RM, AGMver, and AGMsab during OROV infection (**A**). d, day. Ingenuity pathway analysis of DEGs (**B**). MHC, major histocompatibility complex; PI3K, phosphatidylinositol 3-kinase; TGF-β, transforming growth factor–β. Principal components (PC) analysis of plasma analytes. Dot sizes indicate time postinfection (**C**). Longitudinal levels of IP-10 (**D**) and sCD14 (**E**) in PTMs (red), RMs (open), AGMvers (blue), and AGMsabs (green). Error bars correspond to SD. ***P < 0.001 by paired *t* test.

We used flow cytometry (leukocyte frequencies and phenotypes), NanoString analysis of leukocytes (transcriptional analysis), and cytokine bead array and enzyme-linked immunosorbent assay (ELISA) of plasma analytes (soluble inflammatory mediator analysis) to explore the innate and adaptive immune responses elicited during OROV infection (*22*). Longitudinally obtained samples from peripheral blood, BAL, peripheral lymph nodes, and gastrointestinal tract were studied. NanoString analysis of peripheral blood mononuclear cells (PBMCs) revealed many differentially expressed genes (DEGs) associated with type I IFN responses during peak OROV replication in PTM, RM, AGMver, and AGMsab (Fig. 2A). Consistent with innate immunity to RNA viruses, we found up-regulation of IFN responses in PBMCs (Fig. 2A). Differential gene expression was not consistently seen among lymphocytes from other anatomic sites studied. Pathway analysis of DEGs highlighted up-regulation of gene families associated with both the adaptive and innate arms of the immune system, highlighting major inflammation corresponding to OROV infection (Fig. 2B).

To further explore inflammatory responses elicited by OROV infection, we next measured levels of several proinflammatory mediators in longitudinally sampled plasma using cytokine bead arrays and ELISAs to measure 25 analytes. Principal components analysis of these analytes revealed that they were unaltered throughout infection of all four NHP species (Fig. 2C). However, consistent with the NanoString analysis, plasma levels of the type I IFN–responsive gene IP-10 increased concurrent with peak OROV RNA (Fig. 2D). Consistent with only low levels of OROV RNA being found in fecal samples, we did not find elevated levels of soluble CD14 (sCD14; Fig. 2E), suggesting that microbial translocation is not abundant during OROV infection (*23*–*25*). Thus, wide-spread OROV replication throughout the body does not seem apparent.

**Fig. 3.**
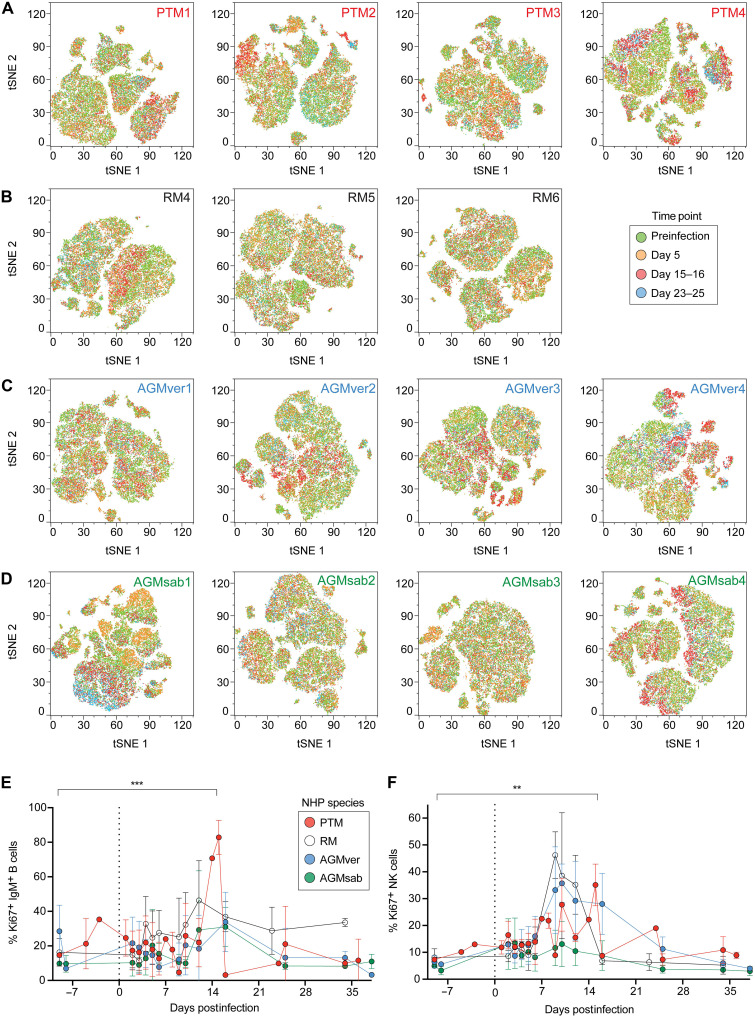
Peripheral blood lymphocyte phenotypes after OROV infection in vivo. Flow cytometric tSNE analysis of longitudinally sampled PBMCs from OROV-infected PTM (**A**), RM (**B**), AGMver (**C**), and AGMsab (**D**). Longitudinal expression of Ki67 among IgM^+^ B cells (**E**). Longitudinal expression of Ki67 among NK cells (**F**) in PTMs (red), RMs (open), AGMvers (blue), and AGMsabs (green). Error bars correspond to SD. **P < 0.01 and ***P < 0.001 by paired *t* test.

We also examined changes in peripheral blood leukocyte populations using spectral cytometry to simultaneously measure the expression of phenotypic markers associated with T cells, B cells, natural killer (NK) cells, and monocytes on PBMCs sampled longitudinally throughout infection (antibodies listed in table S1). t-distributed stochastic neighbor embedding (tSNE) analysis revealed major clustering differences in PTM (Fig. 3A), RM (Fig. 3B), AGMver (Fig. 3C), and AGMsab (Fig. 3D). We noticed cluster differences attributed to the proliferation of CD20^+^ cell (B cell) and NKG2a^+^ cell (NK cell) subsets. In OROV-infected AGMs, CD4^+^ T cells that down-regulate CD4 comprise a large population of CD3^+^ T cells (*26*). During primary infection, upward of 80% of immunoglobulin M–positive (IgM^+^) B cells entered the cell cycle (Fig. 3E), with the highest frequencies of IgM^+^ B cells entering the cell cycle during OROV infection of macaques as compared to AGMs (Fig. 3E). We also found significant proliferation of NK cells during OROV replication (Fig. 3F), which has also been observed during ZIKV infection (*27*). CD4^+^ or CD8^+^ T cell proliferation was less evident (fig. S2, A and B)

### Adaptive immune responses

**Fig. 4.**
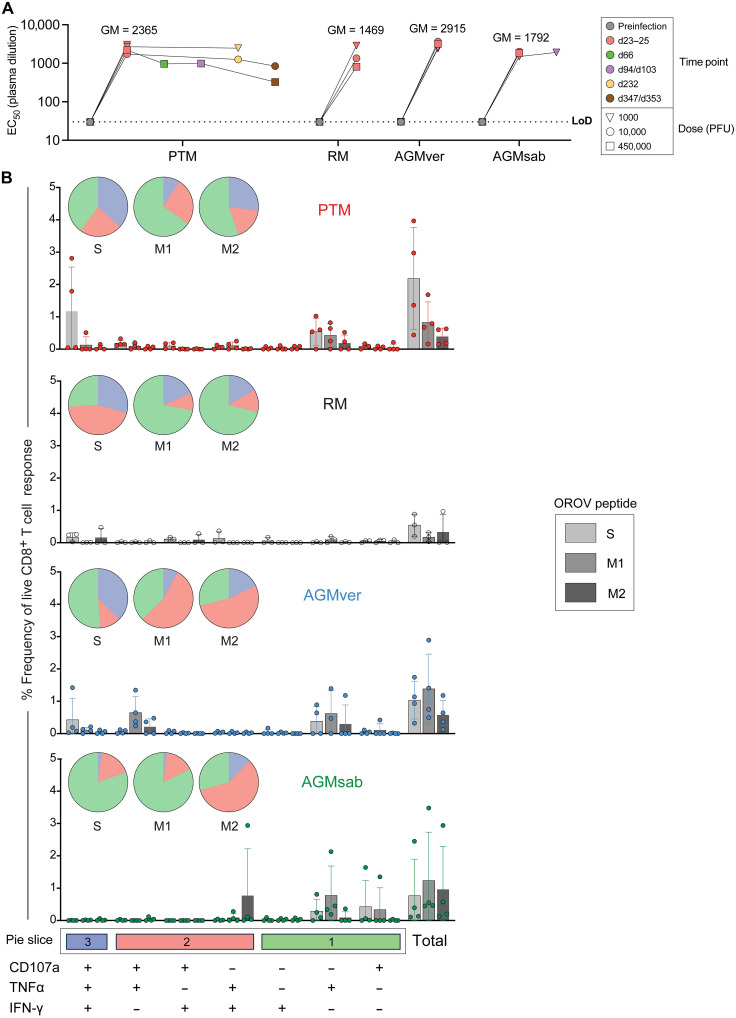
Adaptive immune responses. Longitudinal plasma levels of neutralizing antibodies against OROV in PTMs, RMs, AGMvers, and AGMsabs. Shapes indicate inoculum dose (**A**). Frequencies of CD8^+^ T cells producing combinations of IFN-γ, tumor necrosis factor–α (TNFα), and CD107a after stimulation with OROV peptides spanning the S and M genes (**B**). Error bars correspond to SD and geometric means (GMs) are given in (A). Horizontal line represents limit of detection.

Given the high level of IgM^+^, naïve B cell expansion concomitant with the peak of OROV replication in vivo (Fig. 3), it was possible that these B cells were responding to OROV directly. To measure neutralizing antibody titers against OROV, we developed an in vitro OROV neutralization assay and longitudinally measured the plasma dilution at which 50% of infections were neutralized (EC_50_) (*28*). OROV infection of RMs, PTMs, AGMvers, and AGMsabs elicited neutralization titers between 1000 and 10,000 EC_50_ (median, 2191) by 25 days postinfection (Fig. 4A). We followed plasma samples into the recovery phase in three PTMs and, expectedly, found neutralizing titers dropped by ~4-fold but were still >100 EC_50_ for as long as 1 year postinfection.

We also examined whether T cell responses are elicited in OROV-infected animals. Using 15–amino acid peptide pools, overlapping by 11 amino acids and spanning the M (two pools, M1 and M2) and S genes of OROV, we stimulated OROV-specific T cells ex vivo and measured multiple effector functions by flow cytometry in PTM, RM, AGMver, and AGMsab (Fig. 4B) (*29*, *30*). At 25 days post–OROV infection, we observed detectable frequencies (median, 0.6%) of CD8^+^ T cells that responded to M and S peptides, with higher frequencies of polyfunctional T cells recognizing S peptides compared to M peptides 25 days post–OROV infection. Collectively, these data suggest that primary infection may lead to the induction of immune responses that could offer some protection against subsequent infections.

### Cells that harbor OROV RNA in vivo

**Fig. 5.**
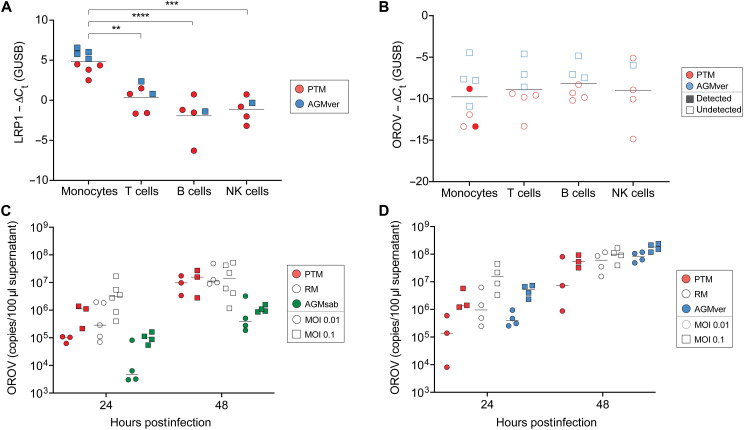
Cells that are infectable with OROV. Levels of LRP1 (**A**) and OROV (**B**) RNA in flow-cytometrically isolated monocytes, T cells, B cells, and NK cells from peripheral blood of PTM and AGMver infected with OROV for 4 to 5 days. OROV RNA levels after in vitro infection of monocyte-derived macrophages (MDMs) (**C**) or fibroblasts (**D**) from PTM, RM, and AGMsab after 24 and 48 hours in PTMs (red), RMs (open), AGMvers (blue), and AGMsabs (green). Filled and open shapes represent detected and undetected OROV RNA in cells in (B), respectively. Shapes indicate infection dose in (C) and (D). Error bars correspond to SD. **P < 0.01, ***P < 0.001, and ****P < 0.0001 by two-way analysis of variance (ANOVA). Horizontal lines represent median.

Given the high levels of OROV RNA detected in plasma (Fig. 1A), we explored whether leukocyte subsets may be targets of OROV infection in vivo. The low-density lipoprotein receptor–related protein (LRP1) is a receptor for OROV (*31*). We measured its expression levels by reverse transcription polymerase chain reaction (RT-PCR) in flow-cytometrically sorted populations of lymphocytes from peripheral blood (Fig. 5A). Monocytes expressed higher levels of LRP1 mRNA than any other population of lymphocytes in peripheral blood, but LRP1 mRNA was also detectable in T cells, B cells, and NK cells (Fig. 5A). To determine infection status of different cell types, we flow-cytometrically isolated lymphocyte subsets at 4 to 5 days post–OROV infection. We found low but detectable levels of OROV RNA in peripheral blood monocytes of only two animals, with other lymphocyte subsets having undetected OROV RNA (Fig. 5B). Given the high plasma viremia and low frequency of OROV RNA^+^ monocytes, it is highly likely that nonhematopoietic cells are infected by OROV in vivo and peripheral blood leukocytes are not major target cells. We were not able to isolate sufficient numbers of tissue-resident macrophages for similar analyses.

### Mechanisms of NHP species–specific OROV susceptibility

The antiviral protein tripartite motif protein (TRIM5α) has been implicated as a determinant of primate species susceptibility to infection with viruses. The PTM genome encodes a TRIM5–cyclophillin A fusion protein that changes the specificity of viral species recognition by TRIM5α and is thought to render PTM more susceptible to certain viral infections including some simian immunodeficiency viruses and flaviviruses (*32*–*37*). As PTMs had high levels of OROV replication in vivo (Fig. 1), we sought to determine whether TRIM5α has inhibitory potential toward OROV using in vitro infections of a validated HAP1 cell line gene edited to delete TRIM5α (TRIM5 knockout) (fig. S3). OROV replication was not increased in TRIM5α knockout cells compared to the parental cell line (fig. S3). Thus, TRIM5α is unlikely to contribute to the different replication kinetics observed in vivo.

We next sought to explore whether primary cells were infectable by OROV in vitro, which may present opportunities for the exploration of cellular mechanisms of viral replication and screening of antiviral compounds (*38*). Peripheral blood monocytes from PTMs, RMs, and AGMsabs were differentiated into monocyte-derived macrophages (MDMs) and infected with 0.01 or 0.1 OROV multiplicities of infection (MOIs) (Fig. 5C). In all cases, high levels of OROV replication were observed at both 24 and 48 hours postinfection with no significant differences between the replication of OROV in MDM from any of the three NHP species we studied. Thus, while monocytes do not seem to be a major target of infection in vivo, ex vivo differentiation into MDM may be a reasonable primary cell model to study aspects of OROV replication, virus host interactions, and screening antiviral compounds ex vivo. We also took small skin biopsies of our different NHP species and cultured fibroblasts followed by infection with either 0.01 or 0.1 OROV MOI and measured OROV RNA levels 24 and 48 hours after infection (Fig. 5D). We found that primary fibroblasts were similarly infectable with OROV compared to MDM with comparable kinetics and levels of OROV RNA being detected. Together, it is clear that primary cell models for OROV infection are feasible.

### Viral reinfection

**Fig. 6.**
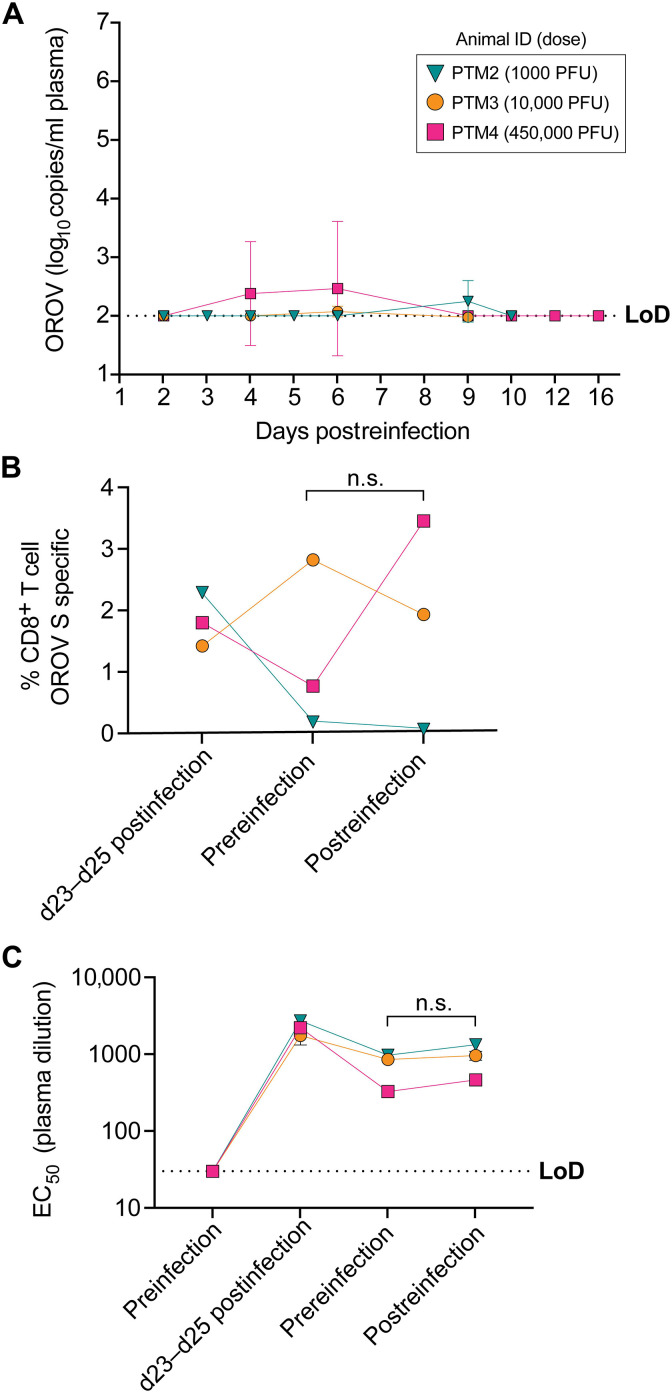
OROV reinfection in vivo. Levels of plasma OROV RNA in three PTM following OROV reinfection after >300 days of initial infection (**A**). Frequencies of CD8^+^ T cells producing combinations of IFN-γ, TNFα, and CD107a after stimulation with OROV peptides spanning the S gene (**B**) and levels of neutralizing antibodies against OROV (**C**). Error bars correspond to SD. n.s., not significant by paired *t* test in (B) and ratio paired *t* test in (C).

The high level of neutralizing antibodies and high frequencies of CD8^+^ T cells elicited against OROV during infection (Fig. 4) suggested that there might be some immunological protection elicited against secondary infection. Therefore, we performed a second infection with OROV in three PTMs that had been previously infected 349, 365, or 524 days previously. Animals were reinfected with 1000, 10,000, or 450,000 median tissue culture infectious doses of OROV (matched to the original infection doses). We measured plasma viremia (Fig. 6A), CD8^+^ T cell responses to the S gene (Fig. 6B), and neutralizing antibody levels (Fig. 6C). Following reinfection, we observed very little detectable OROV RNA for any day we analyzed plasma. When OROV RNA was detected, it was less than 1000 OROV RNA copies (Fig. 6A). Moreover, we found no reliable evidence of boosting either the OROV-specific CD8^+^ T cell frequencies (Fig. 6B) or OROV-specific neutralizing antibody titer (Fig. 6C). Thus, protective immunity was elicited by primary infection and is long lived.

## DISCUSSION

OROV is an arbovirus that is not only thought to be transmitted, predominantly, by the midge but can also efficiently replicate in several mosquito species. OROV has caused more than 500,000 infections since 1950, and there are active areas of virus spread within Central and South America (*7*, *39*, *40*). In 2023 and 2024, high levels of symptomatic OROV infections occurred in South America, and some infections have been reported in Central America and the Caribbean Islands (*12*) and in 19 of 27 federal units in Brazil (*9*). In endemic areas of Brazil, OROV can be detected in 10 to 30% of febrile patients (*9*, *41*, *42*). OROV seropositivity in endemic areas was ~6.5% in early 2025 (*43*). Currently, there are at least two OROV lineages circulating in these areas, and endemic spread is of concern (*13*, *44*). Moreover, OROV infection of individuals traveling from these countries has been observed (*45*–*48*).

Here, we have explored virus replication kinetics and host immune responses following challenge of four species of NHPs with the reemerging bunyavirus OROV. We find that PTM, RM, AGMver, and AGMsab all support viral replication and mount adaptive and innate immune responses to the infection in vivo. We find that the amount of virus required to reliably infect NHPs is 10,000 PFU, which approximates the level of virus that can be found in infected midges (*8*). Given the low numbers of animals within each species that were infected with each viral inoculation, it remains possible that higher doses of inoculation may result in differing viral replication dynamics, but we would speculate these differences would be small. We find considerable innate and adaptive immune responses of all four NHP species to OROV infection. These are similar to those observed against other acute RNA viral infections (*27*). These include up-regulation of type I IFN–associated genes, proliferation of NK cells and B cells, and induction of OROV-specific cellular and humoral immune responses. While our experiments used a laboratory strain of OROV, exploring similar parameters in NHPs infected with primary isolates of OROV should be possible and would be important. PTM and RM seemed to have higher levels of OROV replication and immunity compared to AGMver, with AGMsab having the lowest levels. AGMsabs are frequently used in experiments aimed to explore efficacy of vaccines for respiratory viruses [reviewed in (*15*)]. The lower level of OROV replication in AGMsab may suggest, however, that RM, PTM, or AGMver represents more appropriate and rigorous models for OROV challenge and vaccine and therapeutic studies.

OROV infection is commonly identified with febrile symptomology (*49*). Moreover, many arbovirus symptomologies involve rare neurological invasion, with virus observable within the CSF (*15*). Our work suggests that OROV may be detectable in CSF of infected AGM, although no other NHP species had evidence of OROV within CSF despite high viremia. In addition, unexpectedly, we were able to reliably detect OROV RNA in nasal swabs, suggesting that the virus may replicate in the upper airways and raises the possibility of infection via aerosols. While symptomatic infection is of high clinical importance, the degree to which OROV infections may be asymptomatic is somewhat unclear. The absence of clinical symptomology in all the NHPs we experimentally infected may suggest that robust OROV infection is not sufficient to cause severe symptomology in otherwise healthy macaques and AGMs. The levels of viremia we observe in NHPs approximate those reported in humans (*50*), and, thus, differences in clinical symptomology are unlikely related to differences in levels of viral replication across primate species. High numbers of animals may be required if symptomatic infection is a rare occurrence. For many viral infections where a large frequency of infections are asymptomatic, exploring pathogenesis in NHPs is limiting, given the costs associated with these studies (*15*). Viral replication with subsequent induction of adaptive immune responses following infection on NHPs with viruses that can be pathogenic in humans, without overt symptomology, is a fairly common finding (*15*). Emerging strains of OROV may be more pathogenic than the strain used in our studies, and further work is certainly warranted.

Vertical transmission of OROV in pregnant women (such as ZIKV), has also recently been suggested to potentially occur (*51*–*53*). Modeling vertical transmission in NHPs is challenging, but studies have been performed in pregnant RMs that were infected with ZIKV before infant delivery (*54*–*57*). This suggests that similar experiments of OROV infection in pregnant NHPs might provide an appropriate animal model to explore vertical OROV transmission.

Viral infections of NHPs have been essential to advance antiviral modalities and vaccine candidates against many clinically relevant viruses, including severe acute respiratory syndrome coronavirus 2 (*15*, *58*, *59*). Our data highlight the importance of exploring how virus replicates in different NHP species for subsequent development of antiviral interventions. Together, we believe that RM, PTM, and AGMver are suitable species for exploring interventions that prevent or reduce OROV infection and may also be appropriate for exploring potential increased pathogenicity of clinical isolates of OROV. RM, PTM, and AGMver are also fitting models to explore efficacy of vaccine-induced OROV immunity and antiviral capacity of interventional compounds. Last, our data demonstrating little OROV replication with secondary infection suggest that immunity generated during primary infection is protective against secondary infection, providing hope that vaccine-induced immunity may lead to protection against pathogenic OROV infection in humans.

## MATERIALS AND METHODS

### Nonhuman primates

This study was performed on four adult PTM, four adult AGMver, four adult AGMsab, and six adult RM (Table 1). Full consideration of reducing, replacing, and refining the work without animal models was considered before study initiation. This study was carried out in strict accordance with the recommendations described in the Guide for the Care and Use of Laboratory Animals of the National Institutes of Health, the Office of Animal Welfare, and the US Department of Agriculture (*60*). Animals were monitored twice daily and fed commercial monkey chow, treats, and fruit twice daily by trained personnel. Environmental enrichment was provided in the form of primate puzzle feeders, mirrors, and other appropriate toys.

The National Institute of Allergy and Infectious Diseases (NIAID) Division of Intramural Research Animal Care and Use Program, as part of the National Institutes of Health (NIH) Intramural Research Program, approved all the experimental procedures (animal study protocol LVD26E). The program complies with all applicable provisions of the Animal Welfare Act and other federal statutes and regulations relating to animals. Animals were housed and cared for at the NIH Animal Center, under the supervision of the Association for the Assessment and Accreditation of Laboratory Animal Care–accredited Division of Veterinary Resources and as recommended by the Office of Animal Care and Use Nonhuman Primate Management Plan. Husbandry and care met the standards set forth by the Animal Welfare Act, Animal Welfare Regulations, as well as The Guide for the Care and Use of Laboratory Animals (Eighth Edition). The physical conditions of the animals were monitored daily. Animals were provided continuous access to water and offered commercial monkey biscuits twice daily; fresh produce, eggs, and bread products twice weekly; and a foraging mix consisting of raisins, nuts, and rice thrice weekly. Enrichment to stimulate foraging and play activity was provided in the form of food puzzles, toys, cage furniture, and mirrors or television. All procedures were carried out under ketamine anesthesia by trained personnel under the supervision of veterinary staff, and all efforts were made to maximize animal welfare and minimize animal suffering in accordance with the recommendations of the Weatherall report on the use of NHPs (*61*). Animals were housed in adjoining individual primate cages, allowing social interactions, under controlled conditions of humidity, temperature, and light (12-hour light/12-hour dark cycles). Experimental end points were determined by veterinarian staff on the basis of clinical findings. No animal experienced overt clinical symptomology.

### Viral infection

OROV strain TRVL 9760 (catalog no. VR-1228) was obtained from American Type Culture Collection (Manassas, VA) and grown in a single round of amplification on Vero cells. Viral titer was determined by plaque assay by infecting Vero cell monolayers with serial dilutions of OROV, followed by addition of overlay medium containing 1% methylcellulose. Four days later, cell monolayers were fixed with 4% paraformaldehyde before staining with crystal violet and counting of PFU. Viral stock was deep sequenced using round AB amplification (*62*) (PRJNA1266243). Metavirs (https://github.com/OpenOmics/metavirs) was used to assemble the OROV viral stocks. Briefly, after trimming Illumina adapters, metaspades was used to assemble the reads into viral contigs for further analysis. The nf-core/viralrecon (doi: 10.5281/zenodo.3901628) was used to identify single-nucleotide polymorphisms (SNPs) compared to published sequences of OROV, and bcftools and snpeff were used to call any mutations that occur within the viral stock. We found SNPs frequently reported in bunyaviruses at position 346 of the M gene (*63*). Our challenge stock contained aspartic acid at position 346. Otherwise, SNPs were synonymous. Different doses of viral inoculum were used to infect NHPs subcutaneously (inoculation doses in Table 1).

### Neutralization assay

Neutralizing activity of plasma was determined by focus reduction neutralization test. Serial dilutions of plasma were incubated with ~150 focus-forming units of OROV for 1 hour at 37°C. Immune complexes were then added to Vero cell monolayers and incubated for 1 hour at 37°C, followed by addition of overlay medium containing 1% methylcellulose. Twenty hours postinfection, cells were fixed with 4% paraformaldehyde, permeabilized, and stained for infection foci with an anti-OROV monoclonal antibody (VPS-170). Antibody dose-response curves were analyzed using nonlinear regression analysis (with a variable slope) (GraphPad Software). Data are expressed as the plasma dilution required to reduce infection by half (EC_50_).

### Flow cytometry

Cells were isolated from peripheral blood by density centrifugation. Cells were stained with LIVE/DEAD amine-reactive viability dye (Thermo Fisher Scientific) and a panel of surface antibodies (table S1) for 20 min at 4°C. For sorting, cells were washed in phosphate-buffered saline (PBS) and resuspended in complete RPMI 1640 with 10% fetal bovine serum (FBS) for sorting. For phenotyping, cells were incubated with permeabilization diluent/concentrate mix from the FoxP3/Transcription Factor Staining Buffer set (Invitrogen) for 40 min at 4°C, followed by an addition of intracellular antibodies (table S1) for 20 min at 4°C. Cells were washed with the wash buffer from the buffer set and resuspended in 1% paraformaldehyde before flow cytometry. For T cell peptide stimulation, cells were incubated with Brefedin A (1 μg/ml; Invitrogen), Golgi stop (1 μl/ml; BD Biosciences), and CD107a PE (BioLegend), followed by a 16-hour incubation with 5 μl from the appropriate peptide pool or 5 μl of dimethylsulfoxide (DMSO) as a control. Peptide pools of 15 nucleotide oligomers with an 11–amino acid sequence overlap spanning the S and M OROV genes were synthesized by KareBay Biochem and resuspended in DMSO for a final concentration of 400 μg/ml of each peptide. The peptides for the M gene were resuspended into two pools with M1 covering OROV Gn and NSm, and M2 primarily covering OROV Gc. Following the overnight incubation, cells were washed with PBS and stained as described above.

For each population, a minimum of 10,000 cells were sorted using a FACSymphony S6 cell sorter (BD Biosciences), or a minimum of 50,000 cells were analyzed using a Cytek Aurora cytometer (Cytek). Data analyzed using FlowJo 10 (BD Biosciences). tSNE plots were generated on live CD45^+^ cells with 3000 iterations at a perplexity of 50 (*64*).

### RNA extraction and quantification

For RNA quantification of plasma and CSF, RNA was isolated from 200 μl of sample using the plasma/serum RNA purification mini kit (Norgen Biotech) per the manufacturer’s protocol. For RNA quantification of BAL fluid, RNA was isolated from 5 ml of supernatant using the plasma/serum RNA purification maxi kit (Norgen Biotech) per the manufacturer’s protocol.

For RNA quantification of nasal swabs, sampled swabs were placed in 1 ml of viral transport medium [gentamicin (100 μg/ml; Gibco), amphotericin B (0.5 μg/ml; Sigma-Aldrich), and 2% FBS in Hank’s balanced salt solution (Sigma-Aldrich). RNA was isolated from 140 μl of the extracted swab medium using the QIAamp viral RNA mini kit (QIAGEN) per the manufacturer’s protocol.

For RNA quantification of fecal samples, 250 mg of aliquots of poop into Precellys tubes (Bertin Corp) were homogenized with 750 μl of buffer RLT (QIAGEN) and 1% 2-mercaptoethanol (Sigma-Aldrich). Samples were spun down at 12,000*g* for 1 min, and 450 μl of the supernatant was used to isolate RNA using the RNeasy mini kit (QIAGEN) per the manufacturer’s protocol.

For RNA quantification of tissues, TRIzol-preserved (Sigma-Aldrich) samples were thawed and treated with 200 μl of chloroform to separate nucleic acid into an aqueous phase. Following separation, Total RNA was isolated from the aqueous phase using the MagMAX-96 total RNA isolation kit (Thermo Fisher Scientific) per the manufacturer’s protocol.

For RNA quantification of PBMCs, cryopreserved aliquots of cells were thawed and homogenized using QIAShredders (QIAGEN). RNA was isolated from the homogenate using the AllPrep DNA/RNA mini kit (QIAGEN) per the manufacturer’s protocol.

RNA concentration and purity (absorbance at 260/280 nm ≥ 1.8) were assessed by spectrophotometer and normalized to 50 to 100 ng/μl in PCR-grade H_2_O. For transcript quantification by NanoString (*22*), preparation, hybridization, and detection of RNA samples were carried out by following the NanoString manufacturer’s instructions (NanoString Technologies) using the nCounter NHP Immunology Panel. RNA input was normalized to 33 ng/μl. Subsequent analyses were performed using the nSolver analysis system (v4.0.70, NanoString Technologies). NanoString-generated reads were normalized to internal positive and negative controls and housekeeping genes. NanoString-quantified transcripts were further ascribed to canonical gene expression pathways by ingenuity pathway analysis (v01-19-02, QIAGEN) (*65*).

cDNA was generated using the SuperScript III reverse transcriptase kit (Thermo Fisher Scientific) using random hexamers (Invitrogen). OROV RNA was amplified using forward primer 5′-TACCCAGATGCGATCACCAA-3′, reverse primer 5′-TTGCGTCACCATCATTCCAA-3′, and probe 5′-6-FAM-TGCCTTTGGCTGAGGTAAAGGGCTG-BHQ_1-3′ (*66*). Quantification was determined using serial dilutions of a well characterized plasmid we developed to contain the OROV amplicon (*19*). Samples were assessed in duplicate on the Applied Biosystems QuantStudio 3 (Thermo Fisher Scientific), using the recommended TaqMan Fast Advanced cycling parameters.

For individual transcript quantification, cDNA was generated as described above. Quantitative RT-PCR (qRT-PCR) for *OROV* and *LRP1* were performed using transcript-specific gene expression assays (Thermo Fisher Scientific) with TaqMan Fast Advanced Master Mix and were normalized to housekeeping gene (monkey) β-glucuronidase (mGusB) using a custom-designed mGusB TaqMan gene expression assay (Thermo Fisher Scientific). Custom mGusB primers and probe are as follows: forward primer, 5′-CTCATTTGGAATTTTGCCGATT-3′; reverse primer, 5′-CCGAGTGAAGATCCCCTTTTTA-3′; probe, 5′-TGAACAGTCACCGACGAGAGTGCTGG-3′. Custom LRP1 primers are as follows: forward primer, 5′-AGTCGTCTCTGCAGACTTGC-3′; reverse primer, 5′-TGCGTTCTTGAAGGAGCCAT-3′. Relative transcript measurement was assessed in triplicate on the Applied Biosystems QuantStudio 3 System (Thermo Fisher Scientific), using the recommended TaqMan fast advanced cycling parameters.

### Plasma biomarker analysis

Plasma samples were screened for biomarkers using the MILLIPLEX Non-Human Primate Cytokine Magnetic Bead premixed panel (Sigma-Aldrich) reading with the Bio-Plex 200 system (Bio-Rad) following the recommended settings per the manufacturer’s protocol. In addition, samples were also tested for sCD14 and IP-10 using the human Quantikine CD14 ELISA kit and human Quantikine CXCL10/IP-10 ELISA kit (R&D Systems). Plates were read at 450 and 540 nm for background correction. Values calculated as less than zero were reported as zero. Principal components analysis plots were generated using GraphPad Prism 10.0 using the recommended settings.

### In vitro infections

#### TRIM5 knockout infections

HAP1 wild-type and TRIM5 knockout cells were plated at 1 × 10^5^ cells per well in a 24-well plate and infected with OROV (MOI = 0.001 and 0.01) for 1 hour at 37°C the following day as previously reported (*37*). Cells were washed once with PBS and incubated with HAP1 complete medium, and supernatant was collected 24 and 48 hours postinfection. Plaque assays were performed to determine viral infectivity levels. Vero cells were plated at 1 × 10^5^ cells per well in a 48-well plate and infected with 100 μl of serially diluted virus supernatant for 1 hour before addition of a 1.5% *O*-carboxymethylcellulose in minimum essential medium overlay for 3 days before fixation with 10% formalin and staining with crystal violet. Plaques were counted and virus titer expressed as PFU per milliliter.

#### Primary MDMs and fibroblast infections

PBMCs were isolated from healthy, simian immunodeficiency virus–negative animals by density gradient centrifugation. Macrophages were differentiated from monocytes as previously described (*38*). Cells were resuspended at 3 × 10^6^/ml in RPMI 1640 containing 2 mM l-glutamine (Gibco), penicillin (100 U/ml; Gibco), streptomycin (100 μg/ml; Gibco), 0.1 mM sodium pyruvate (Gibco), 1% nonessential amino acids (Gibco), 50 μM 2-mercaptoethanol (Gibco), and 1% human AB serum (Sigma-Aldrich) and allowed to adhere to tissue culture plates overnight. After 24 hours, cells were washed twice with RPMI 1640 to remove most of the nonadherent cells and cultured for an additional 5 days in the culture medium described above supplemented with M-CSF (20 ng/ml; Peprotech) and interleukin-1β (10 ng/ml; Peprotech).

Skin biopsies were rinsed in 70% ethanol (to reduce bacterial burden) and then PBS. Tissue was minced with a scalpel and incubated with 2 ml of 0.28 Wu/ml of Liberase in Dulbecco’s modified Eagle’s medium (DMEM) in a six-well plate for 1 hour. Four milliliters of pLF medium [DMEM with sodium pyruvate, L-glutamine, 10% FBS, penicillin (100 IU/ml), streptomycin (100 μg/ml), gentamicin (30 μg/ml), amphotericin (15 ng/ml), 1× 2-mercaptoethanol, and 1× nonessential amino acids] was added and incubated overnight. Five hundred microliters of EDTA solution (2.5 mM EDTA in PBS with 2% FBS) was added, mixed, and incubated at 37°C for 5 min. The tissue mixture was triturated using a P1000, passed through 100-μm cell strainer, and washed with ~20 ml of sterile PBS. Cells were pelleted by centrifuging at 1700 rpm for 4 min, resuspended in 3 ml of pLF medium, and plated in new six-well plate. Medium was changed every other day, and cells were replated upon reaching confluency

Macrophages or fibroblasts were plated at 1.5 × 10^5^ per well in 12-well plates and infected the next day with an MOI of 0.1 or 0.01 for 1 hour in 350 μl of medium at 37°C. Wells were then washed twice with RPMI 1640 (macrophages) or DMEM (fibroblasts) and incubated with 1 ml of medium. One hundred forty microliters of supernatant was removed at 24 and 48 hours, and viral RNA was extracted using a QIAamp viral RNA kit per the manufacturer’s instructions.

### Statistics

Unpaired or paired, one- and two-way *t* tests were used in statistical analyses of plasma biomarkers, single-parameter lymphocyte phenotype and function, viral load, single transcript qRT-PCR assessments, and reinfection neutralizing antibody and peptide stimulation responses (Prism v9.0, GraphPad Software).
