# Supplementary material for: Oropouche virus efficiently replicates and is immunostimulatory in vivo in nonhuman primate species
Source: Sci Adv. 2025 Sep 17;11(38):eadx9405. doi: 10.1126/sciadv.adx9405 (PMC12442868; doi:10.1126/sciadv.adx9405)
Supplement: Supplementary file 1 — Figs. S1 to S3 Table S1 Legends for data S1 to S9 [file sciadv.adx9405_sm.pdf]

Science Advances
AAAS

Supplementary Materials for

Oropouche virus efficiently replicates and is immunostimulatory in vivo in nonhuman primate species

Debra S. Yee *et al.*

Corresponding author: Jason M. Brenchley, jbrenchl@mail.nih.gov

Sci. Adv. **11**, eadx9405 (2025)
DOI: 10.1126/sciadv.adx9405

The PDF file includes:

Figs. S1 to S3
Table S1
Legends for data S1 to S9

Other Supplementary Material for this manuscript includes the following:

Data S1 to S9

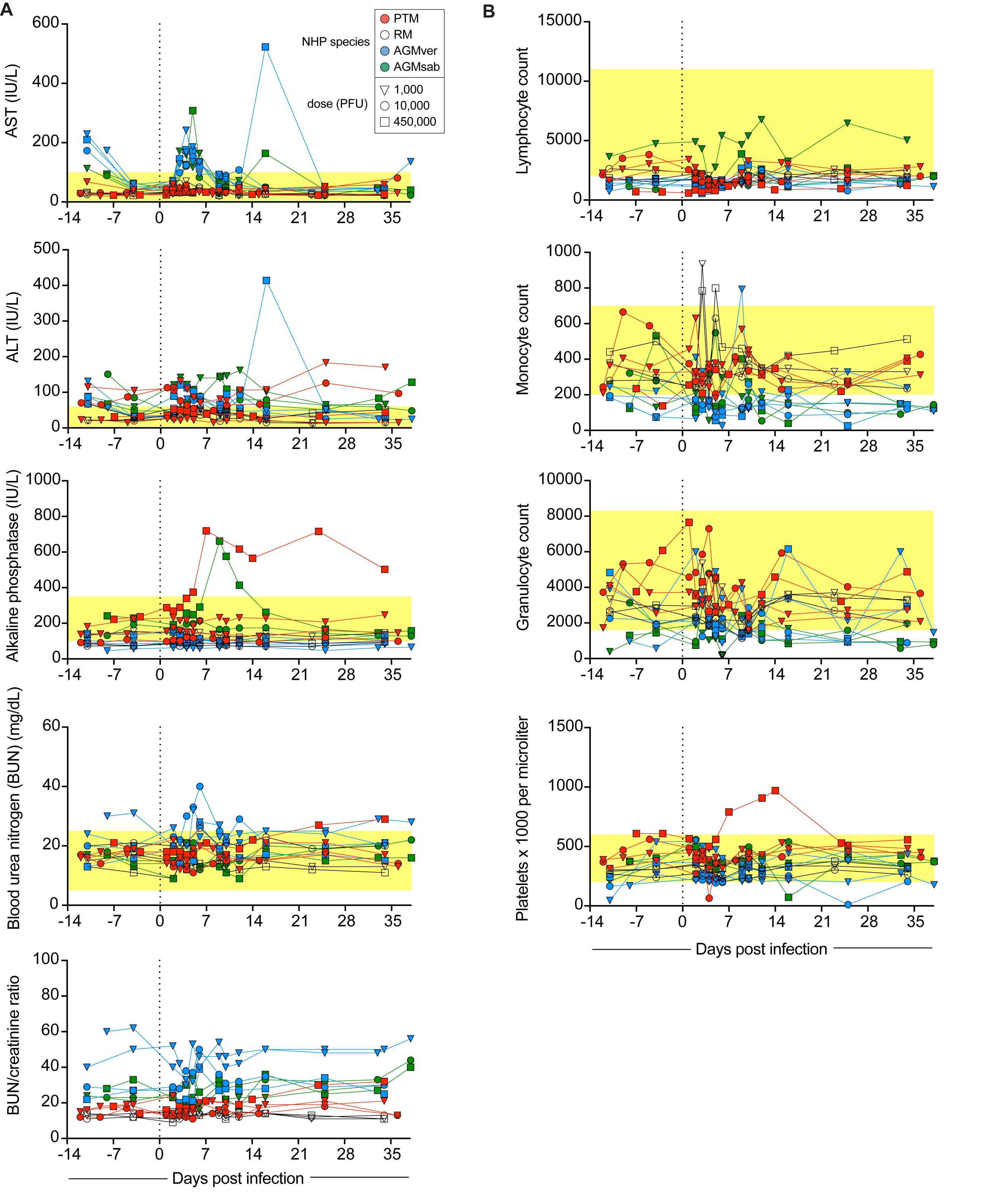

Supplemental Figure S1. Clinical observations after OROV infection. Longitudinal blood chemistry panels monitoring aspartate aminotransferase (AST), alanine aminotransferase (ALT), alkaline phosphatase, blood urea nitrogen (BUN), and BUN/creatinine ratios (A). Longitudinal complete blood counts of lymphocytes, monocytes, granulocytes, and platelets (B) in pigtail macaques, PTM (red), rhesus macaques, RM (open), vervet African green monkeys, AGMver (blue), and sabeus African green monkeys, AGMsab (green). Highlighted yellow regions correspond to standard blood ranges.

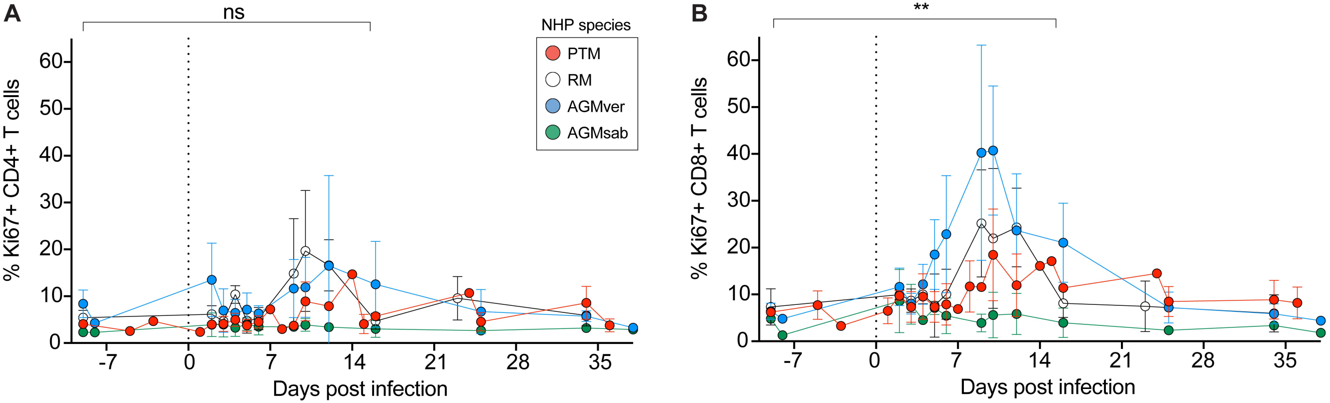

Supplemental Figure S2. Longitudinal proliferation of peripheral blood CD4+ and CD8+ T cells after OROV infection. Ki67 expression among CD4+ T cells (A) and among CD8+ T cells (B) in pigtail macaques, PTM (red), rhesus macaques, RM (open), vervet African green monkeys, AGMver (blue), and sabeus African green monkeys, AGMsab (green). Error bars correspond to standard deviation. ** P<0.01, ns not significant by paired t test.

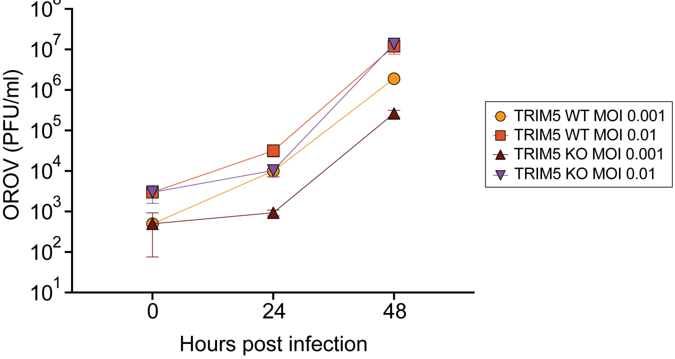

Supplemental Figure S3. OROV PFU after *in vitro* infection of TRIM5α expressing and knockout HAP1 cells.

Phenotyping

Manufacturer	Catalog Number	Fluorophore	Marker	Clone
BD Biosciences	552852	PerCP-Cy5.5	CD3	G20-127
BD Biosciences	555787	PE	IgG	G18-145
BD Biosciences	556026	FITC	Ki67	B56
BD Biosciences	560459	V450	FoxP3	259D/C7
BD Biosciences	560734	APC-H7	CD20	2H7
BD Biosciences	561288	AlexaFluor700	CD45	D058-1283
BD Biosciences	562296	PE-CF594	CD28	CD28.2
BD Biosciences	562721	BV605	CD11b	ICRF44
BD Biosciences	612936	BUV496	CD4	SK3
BD Biosciences	740998	BV786	IgM	G20-127
BD Biosciences	746912	BV750	HLA-DR	G46-6
Beckman Coulter	B10246	PECy7	CD159a/NKG2a	Z199
Biolegend	302044	BV711	CD16	3G8
Biolegend	305610	PECy5	CD95	DX2
Biolegend	344730	BV650	CD8	RPA-T8
Jackson ImmunoResearch	109-135-011	APC	IgA	polyclonal

Peptide Stimulation

Manufacturer	Catalog Number	Fluorophore	Marker	Clone
BD Biosciences	552852	PerCP-Cy5.5	CD3	G20-127
BD Biosciences	551383	APC	IL2	MQ1-17H12
BD Biosciences	557745	PECy7	CD69	FN50
BD Biosciences	560371	BV450	IFNg	B27
BD Biosciences	561277	AlexaFluor700	Ki67	B56
BD Biosciences	612936	BUV496	CD4	SK3
Beckman Coulter	6607111	ECD	CD28	CD28.2
Biolegend	301038	BV570	CD8	RPA-T8
Biolegend	305610	PECy5	CD95	DX2
Biolegend	328608	PE	CD107a	H4A3
Biolegend	359418	BV605	CCR4	L291H4
Biolegend	502948	BV785	TNFa	MAb11
Biolegend	512328	BV711	IL17	BL168
Invitrogen	11-1038-42	FITC	CD103	B-Ly7
Invitrogen	47-1548-42	APC-eFluor780	CD40L	24-31

Supplemental Table S1. Antibodies used in flow cytometry staining.

Data S1. Excel data file of OROV viral RNA copies for Figure 1

Data S2. Excel data file of Nanostring counts, Ingenuity pathway z-score outputs, cytokine bead array mean fluorescence intensities, and ELISA data for Figure 2

Data S3. Excel data file of Ki67+ IgM+ B cell and NK cell populations for Figure 3

Data S4. Excel data file of FRNT values and peptide stimulation frequencies from gated populations for Figure 4

Data S5. Excel data file of qPCR data of LRP1 or OROV copies for Figure 5

Data S6. Excel data file of re-infection OROV copies, stimulation frequencies, and FRNT values for Figure 6

Data S7. Excel data file of total blood chemistry results and complete blood counts for OROV+ NHPs for Supplemental Figure 1

Data S8. Excel data file of Ki67+ CD4+ and CD8+ cell populations for Supplemental Figure 2

Data S9. Excel data file of OROV virus titer counts from HAP1 and TRIM5 knockout plaque assays for Supplemental Figure 3
